# Systematic proteomics analysis of lysine acetylation reveals critical features of placental proteins in pregnant women with preeclampsia

**DOI:** 10.1111/jcmm.16997

**Published:** 2021-10-26

**Authors:** Yu Shangguan, Yinglan Wang, Wei Shi, Ruonan Guo, Zhipeng Zeng, Wenlong Hu, Wanxia Cai, Qiang Yan, Yong Xu, Donge Tang, Yong Dai

**Affiliations:** ^1^ Clinical Medical Research Center Guangdong Provincial Engineering Research Center of Autoimmune Disease Precision Medicine Shenzhen Engineering Research Center of Autoimmune Disease The Second Clinical Medical College of Jinan University, The First Affiliated Hospital of Southern University of Science and Technology Shenzhen People's Hospital Shenzhen China; ^2^ Guangxi Key Laboratory of Metabolic Disease Research Nephrology Department 924st Hospital Guilin China; ^3^ College of Life Science Guangxi Normal University Guilin China

**Keywords:** angiogenesis, complement and coagulation cascades, immune system, lysine acetylation, preeclampsia, proteomics

## Abstract

Preeclampsia (PE) is a dangerous hypertensive disorder that occurs during pregnancy. The specific aetiology and pathogenesis of PE have yet to be clarified. To better reveal the specific pathogenesis of PE, we characterized the proteome and acetyl proteome (acetylome) profile of placental tissue from PE and normal‐term pregnancy by label‐free quantification proteomics technology and PRM analysis. In this research, 373 differentially expressed proteins (DEPs) were identified by proteome analysis. Functional enrichment analysis revealed significant enrichment of DEPs related to angiogenesis and the immune system. COL12A1, C4BPA and F13A1 may be potential biomarkers for PE diagnosis and new therapeutic targets. Additionally, 700 Kac sites were identified on 585 differentially acetylated proteins (DAPs) by acetylome analyses. These DAPs may participate in the occurrence and development of PE by affecting the complement and coagulation cascades pathway, which may have important implications for better understand the pathogenesis of PE. In conclusion, this study systematically analysed the reveals critical features of placental proteins in pregnant women with PE, providing a resource for exploring the contribution of lysine acetylation modification to PE.

## 1 INTRODUCTION

Preeclampsia (PE) is a placenta‐induced hypertensive disorder of pregnancy that affects 3–5% of pregnancies, and it is one of the main causes of morbidity and mortality in mothers and foetuses.^1^, ^2^ When left untreated, PE can lead to significant heart, liver, kidney, brain and lungs dysfunction in pregnant women, as well as a series of foetal complications (such as neonatal growth restriction, neonatal respiratory distress syndrome, autism spectrum disorder, cerebral palsy or even death).^2^, ^3^ Some scholars have proposed a two‐stage model of PE: stage 1 is poorly perfused placenta in the first trimester, wherein the failure of trophoblast invasion leads to dysfunctional spiral artery remodelling, and hypoxia may cause oxidative stress and imbalance of reactive oxygen species in the placenta of PE; stage 2 is a maternal syndrome in the last second and third trimesters characterized by angiogenesis imbalance, inflammatory cytokines and immune cell alterations.^4^, ^5^ The placental tissue is directly exposed to the uterine decidua, which is involved in maternal–foetal immune tolerance and pregnancy maintenance.^6^ Although its exact pathogenesis and triggering conditions are still not fully clarified, it is widely believed that the placenta plays a central role in PE formation.^7^ The most common cause of PE includes placental hypoxia, oxidative stress, disturbed angiogenesis, increased proinflammatory cytokines, complement dysregulation and endothelial dysfunction.^5^, ^8^, ^9^ Recent research has found that the immune system and mitochondrial dysfunction play an essential role in the pathophysiology of PE.^10^, ^11^ Interestingly, hypoxia leads to placental chromatin modification, which reduces placental histone acetylation and placental acetyl‐CoA, suggesting that acetylation modification may be critical to the pathogenesis of PE.^3^

Proteins posttranslational modifications (PTMs) play a crucial role in regulating various biological processes, which can modulate the structure, activity and function of proteins by introducing new functional groups.^12^, ^13^ Lysine acetylation (Kac) is an essential posttranslational modification discovered in histones in the 1960s.^14^ This modification regulates many biological functions (such as chromatin remodelling, gene expression regulation, protein stability, cytoskeletal dynamics and cellular metabolic state) and is closely linked to cardiovascular diseases.^15^, ^16^, ^17^ However, there is still a lack of available studies on the role of Kac modification in PE. Previous researchers have used iTRAQ proteomics to identify differentially expressed proteins (DEPs) in PE and normal pregnancy placental tissues to look for potential biomarkers of PE.^6^ Quantitative analysis and PTMs of proteins are essential for understanding biological complexity.^12^, ^18^ To better understand the pathogenesis of PE, we integrated proteome and acetyl proteome (acetylome) systematically to analyse the vital features of placental proteins in pregnant women with PE and provided a landscape of acetylation in PE, which may contribute to developing valuable novel biomarkers for PE.

## 2 MATERIALS AND METHODS

### 2.1 Sample collection

In this study, six pregnant women with PE and six normal‐term pregnant women admitted to the Department of Obstetrics and Gynecology, Shenzhen People's Hospital, from February 2020 to July 2020, were recruited and divided into the PE group and NC group. The inclusion criteria of the PE group were ACOG gestational hypertension and preeclampsia guidance.^19^ All the pregnant women were without infection, coronary heart disease, chronic kidney disease, tuberculosis or other diseases affecting placental protein content. The study was approved by the Medical Ethics Committee of Shenzhen People's Hospital. All pregnant women signed written informed consent before inclusion in the study. Placental tissue near the maternal side's central area was collected immediately in an aseptic environment after delivery. Calcification, necrosis and vascular areas were excluded from sampling. The residual maternal blood and amniotic fluid were washed with PBS, and the excess water was removed with sterile filter paper. Finally, the tissue samples were put into centrifuge tube and transferred to −80°C refrigerator for further processing.

### 2.2 Sample processing

Protein extraction and trypsin digestion were carried out using the published method.^20^ Placental tissue samples were removed from a −80°C refrigerator and ground into cell powder with liquid nitrogen. After that, a four‐fold volume of lysis buffer (1% Triton‐100, 1% protease inhibitor, 3  μM TSA and 50  mM NAM) was added to each centrifuge tube containing cell powder. Then, the suspension was sonicated on ice with a high‐intensity ultrasound processor. The remaining debris was removed by centrifugation for 10  min (12000  *g* at 4℃). Subsequently, we used a BCA protein assay kit (Beyotime) to determine the protein concentration in the supernatant. After treatment with trichloroacetic acid (TCA), the protein precipitation was dispersed with 200  mM tetraethylammonium bromide (TEAB) and then digested overnight with trypsin at a ratio of 1:50 (trypsin: protein). Upon addition of dithiothreitol (DTT; final concentration of 5  mM), samples were reduced for 30  min at 56°C, followed by addition of iodoacetamide (final concentration of 11  mM) and incubation for 15  min at room temperature in the dark.

### 2.3 Affinity enrichment of Kac peptides

Compared with the total proteome, the acetylome added an IP enrichment process. Before Kac enrichment, pan anti‐acetyllysine antibody beads (Lot number: PTM‐104, PTM Bio) were prewashed with PBS. The peptides were dissolved in IP buffer (100  mM NaCl, 1  mM EDTA, 50  mM Tris‐HCl, 0.5% NP‐40, pH 8.0), and then the supernatant was incubated with antibody beads at 4°C overnight with mild shaking. After incubation, the antibody beads were washed four times with IP buffer and twice with ddH_2_O. Finally, we used 0.1% trifluoroacetic acid to elute the bound Kac peptides from antibody beads. The eluted fractions after vacuum drying were desalted with C18 ZipTips (Merck Millipore) and then analysed by LC‐MS/MS.^21^

### 2.4 LC‐MS/MS analysis

The peptides from total protein digestion or enrichment of Kac peptides were dissolved in 0.1% formic acid and 2% acetonitrile before being injected into a nanoElute UPLC system (Bruker). For the peptides used for proteome quantification, the gradient elution was set to solvent B (0.1% formic acid and 100% acetonitrile) increase from 6% to 24% over 70  min, 24% to 35% in 14  min, a linear increase to 80% in 3  min and then maintained in 80% for the last 3  min; the flow rate was constant at 450  nL/min.^22^ For the Kac peptides used for acetylome quantification, the gradient elution was set to solvent B increase from 7% to 22% over 40  min, 22% to 30% in 14  min and climbing to 80% in 3  min and then maintained in 80% for the last 3  min; the flow rate was constant at 350 nL/min. Peptides were ionized with 2.0  kV electrospray voltage from a capillary ion source on a timsTOF Pro mass spectrometer (MS) (Bruker Daltonics). Precursor ions were detected in the TOF MS at a resolution of 30000. The data acquisition used the parallel accumulation serial fragmentation (PASEF) mode.^23^ Dynamic exclusion was set to 30  s to reduce repeated sequencing of precursor ions.

### 2.5 Database search

The MaxQuant search engine (v.1.6.15.0) was used to process MS/MS data. 24LC‐MS/MS was searched against the SwissProt database (Homo sapiens, containing 20366  sequences) concatenated with the reverse decoy database.^25^ Trypsin/P was specified as a cleavage enzyme allowing up to four missed cleavages and five modifications per peptide, and the minimum peptide length was set as 7. Cysteine carbamidomethylation was defined as fixed modification; protein N‐terminal acetylation and methionine oxidation were defined as variable modifications. False discovery rate (FDR) thresholds for peptides, proteins and modification sites were adjusted to 1%. These common parameters were used for proteome and acetylome database search analysis. In addition, the identification of the acetylome used the following additional settings: Kac was defined as variable modification and modified peptide score was set at >40.

### 2.6 Parallel Reaction Monitoring analysis

Tissue collection and processing were the same as described above. For the total proteome, the tryptic peptides of each sample were separated on an EASY‐nLC 1000 UPLC system (Thermo Fisher Scientific). Subsequently, the eluted peptides were analysed using Q Exactive™ Plus MS (Thermo Fisher Scientific), with the following parameters: PRM mode, the MS scan range 406–1195  m/z, automatic gain control (AGC) target was set to 3E6 for full MS and 1E5 for MS/MS, the maximum injection time (IT) was 50  ms for full MS and 160  ms for MS/MS, and the isolation window of MS/MS was set to 1.6  m/z. For the acetylome, the enriched Kac peptides were separated on an EASY‐nLC 1200 UPLC system (Thermo Fisher Scientific). Then, the eluted peptides were analysed using Q Exactive™ HF‐X MS (Thermo Fisher Scientific) with the following parameters: PRM mode, MS scan range 430–930  m/z, AGC target was set to 3E6 for full MS and 1E5 for MS/MS, the maximum IT was 50  ms for full MS and 200  ms for MS/MS, and the isolation window of MS/MS was set to 1.4  m/z.^26^, ^27^ For the PRM data analysis, Skyline (v.3.6)^28^ was used to process the obtained MS data.

### 2.7 Bioinformatics and statistical analysis

The differences between PE and NC were calculated using fold change (FC) as a criterion. FC>1.5 and FC<1/1.5 were considered as up‐regulated and down‐regulated proteins, respectively. *p*  <  0.05 was the critical value for identifying DEPs and differentially acetylated proteins (DAPs). Gene ontology (GO) annotation of human proteins was derived from the UniProt‐GOA database (www. http://www.ebi.ac.uk/GOA/). WoLF PSORT (https://wolfpsort.hgc.jp/) can predict the subcellular location of submitted proteins. We used Kyoto Encyclopedia of Genes and Genomes (KEGG) to identify the pathways enriched in DEPs and DAPs, and used Motif‐x software to analyse the sequence model, which contains amino acids at specific position of acetyl 21‐mer in all protein sequences (10 amino acids upstream and downstream of the acetylation site). In addition, the volcano plot, heat map and GO enrichment analysis were generated using PTMCloud online tools (http://www.ptmbiolab.com). The KEGG pathway enrichment network for the DAPs was constructed using the ClueGO plug‐in in Cytoscape.^29^ GraphPad Prism 8  software was used to generate box plots and calculate statistical significance. *p*<0.05 was considered significant.

## 3 RESULTS

### 3.1 Clinical characteristics

The clinical characteristics of women with PE and NC were given in Table 1. We analysed critical biometric data such as maternal age, gestational age, maternal BIM, systolic blood pressure, diastolic blood pressure, 24  h proteinuria and baby birth weight. The results showed no significant difference in maternal age, body mass index (BMI) or baby weight between the two groups (*p *>  0.05). Simultaneously, systolic blood pressure, diastolic blood pressure and 24‐h proteinuria in patients with PE were significantly higher than those in NC.

**TABLE 1:** The clinical characteristics of pregnant women with PE and NC

	NC (*n* = 6)	PE (*n* = 6)	*p*‐value
Maternal age (year)	31.50 ± 3.62	33.00 ± 5.90	0.607
Gestational age (week)	38.75 ± 0.99	35.32 ± 1.66	0.001
BMI (kg/m^2^) Systolic blood pressure (mm Hg)	24.48 ± 1.54 109.50 ± 10.52	26.05 ± 3.72 156.00 ± 21.3	0.360 <0.001
Diastolic blood pressure (mm Hg) 24‐h proteinuria (ng/24h) Baby weight (kg)	73.67 ± 14.61 / 3.02 ± 0.25	104.67 ± 17.34 584.41 ± 469.40 2.39 ± 0.68	0.007 / 0.062

Note

Data were given as arithmetic mean ± standard error.

Abbreviations: BMI, body mass index; NC, normal‐term pregnant women; PE, preeclampsia.

### 3.2 Proteome profiling of PE placental tissues

**FIGURE 1:**
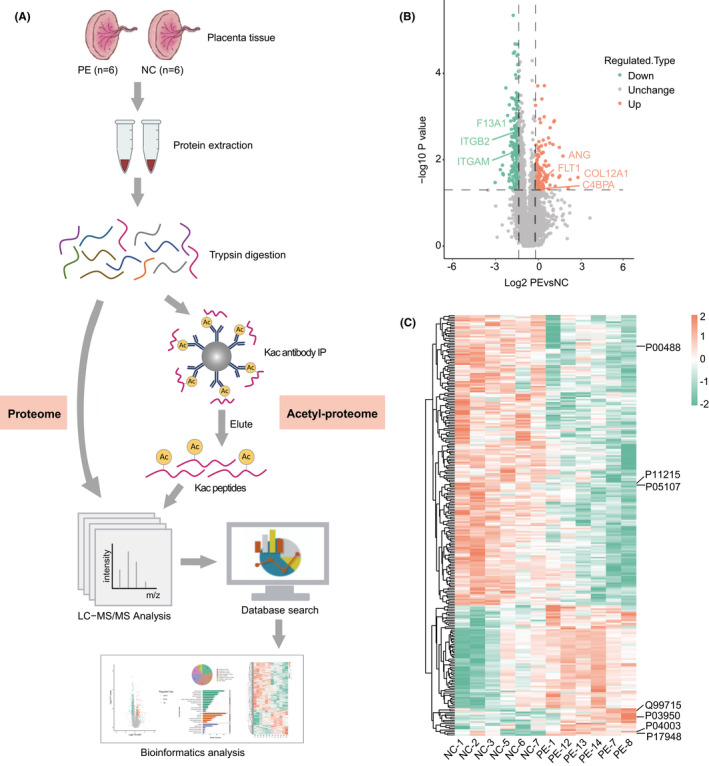
Proteome profiling in placental tissues. (A) Flowchart of the label‐free quantitative proteome and acetyl proteome in placental tissue. (B) Volcano plot of DEPs in placental tissues. (C) Hierarchical clustering of DEPs. The colour scale bar represents the log2 value of the relative proteins expression level between PE and NC (orange: high; green: low). Abbreviations: DEPs, differentially expressed proteins; PE, preeclampsia; NC, normal‐term pregnancy

An overview of the experimental procedure is shown in Figure 1A. A total of 6460 proteins were identified in label‐free quantification proteomics analysis, of which 5457 proteins had a quantifiable level. Among these quantifiable proteins, 373 proteins showed statistically significant changes between PE and NC, including 127 proteins up‐regulated and 246 down‐regulated (Figure 1B). It is noteworthy that up‐regulated proteins include COL12A1, ANG, FLT1 and C4BPA, and the down‐regulated proteins include F13A1, ITGAM and ITGB2 (Figure 1B). Hierarchical clustering analysis of DEPs is depicted in Figure 1C, demonstrating a moderate distinction between NC and PE.

### 3.3 Function enrichment analysis of DEPs

**FIGURE 2:**
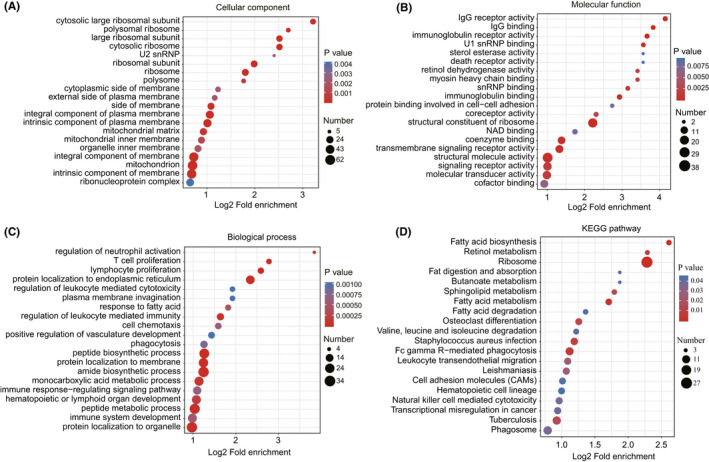
Function enrichment analysis of DEPs between PE and NC placental tissues. Enriched GO terms in the (A) Cellular component, (B) Molecular function and (C) Biological process categories. (D) KEGG pathway enrichment analysis of DEPs

**FIGURE 3:**
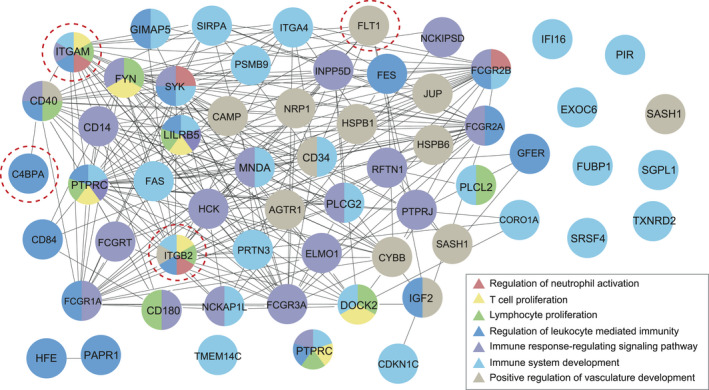
Enriched GO terms in the biological process of selected categories. Circles represent DEPs, and different colours indicate different biological processes term

To further clarify the functions of these DEPs, we performed GO annotation (cellular component, molecular function and biological process) and KEGG pathway enrichment analysis. Cellular component enrichment analysis in Figure 2A revealed that DEPs were significantly enriched in the cytosolic large ribosomal subunit and polysomal ribosome, with the largest number of proteins localized to the mitochondrion. According to molecular function analysis (Figure 2B), DEPs were related to IgG receptor activity, IgG binding and immunoglobulin receptor activity, and most proteins were involved in the structural constituent of ribosome and structural molecule activity. Within the biological process analysis, DEPs were involved in the regulation of neutrophil activation, T‐cell proliferation, lymphocyte proliferation, regulation of leucocyte‐mediated immunity, positive regulation of vasculature development, immune response‐regulating signalling pathway, hematopoietic or lymphoid organ development and immune system development, revealing that DEPs may be connected with immunity and angiogenesis (Figure 2C). It is worth noting that ITGB2, ITGAM and C4BPA were involved in multiple biological processes associated with immunity, and FLT1 was involved in the positive regulation of vascular development (Figure 1B, Figure 3). Furthermore, KEGG pathway analysis revealed that DEPs were enriched within the ribosome, fatty acid biosynthesis, leucocyte transendothelial migration, hematopoietic cell lineage and natural killer cell mediated cytotoxicity pathways (Figure 2D).

### 3.4 Profiles of DAPs and motif analysis of Kac sites in PE placental tissues

**FIGURE 4:**
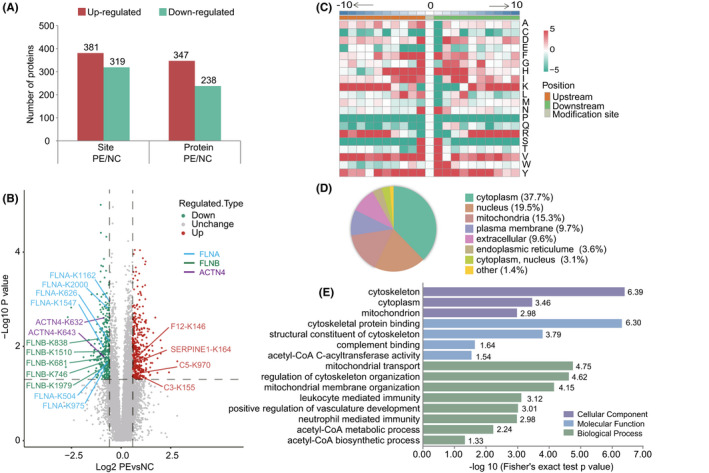
Characteristics of the DAPs. (A) Statistical analysis of DAPs and Kac sites. Red represents up‐regulated, and green indicates down‐regulated. (B) Volcano plot showing the distribution of Kac sites in placental tissues. FLNA, FLNB and ACTN4 have multiple Kac sites. (C) Motif analysis of the amino acid compositions around Kac sites. Red represents enrichment; green represents depletion. (D) Subcellular location information of DAPs in placental tissues. (E) GO enrichment analysis of DAPs between PE and NC placental tissues. Purple rectangle represents cellular composition, blue rectangle represents molecular function and green rectangle represents biological process categories. Abbreviations: DAPs, differentially acetylated proteins

We corrected the quantification values corresponding to the acetylated peptide sites using total proteins quantitation values, which avoided misinterpreting possible positive or negative regulations at the protein level as regulations at the residue acetylation level.^21^ After normalizing against the total quantitative proteins, 381 up‐regulated Kac sites of 347 DAPs and 319 down‐regulated Kac sites of 238 DAPs were identified between PE and NC placental tissues (Figure 4A). Notably, among these DAPs, FLNA, FLNB and ACTN4  have multiple Kac sites (Figure 4B).

Previous studies have reported that the conserved protein sequence motifs are connected with Kac.^30^ To research the common sequence patterns around the Kac sites, Motif‐X was used to analyse the sequence motifs of 10 amino acids flanking the identified Kac sites. The motif enrichment heat map (Figure 4C) showed the enrichment or depletion of all identified Kac sites’ upstream and downstream amino acids. It was observed that phenylalanine (F), histidine (H), valine (V) and tyrosine (Y) tend to be enriched near the Kac site. In contrast, proline (P) and glutamine (Q) tended to be reduced in the proximity of the Kac site. Of note, serine (S) and threonine (T) were significantly enriched at positions −1 to +1, while other positions were reduced. These findings reflected that the amino acids around the Kac site have different preferences. Although some motifs, such as KF, KH and KY, have been found in other species,^13^, ^31^, ^32^ whether these different types of enzymes that regulate acetylation can affect the pathophysiology of PE remains to be further studied.

### 3.5 Subcellular localization and functional enrichment analysis of DAPs

Protein acetylation modification regulates many critical cellular processes and has vital implications for protein transport and cellular localization.^15^ Kac was first discovered in histones.^14^ In addition to histones, acetylated proteins were found in the nucleus, cytoplasm, mitochondria and other cell compartments.^13^ Consistent with previous studies, DAPs were mainly distributed in the cytoplasm, nucleus and mitochondria^31^, ^33^, ^34^ (Figure 4D). Kac widely exists in mitochondria and is closely associated with the regulation of energy generation and fatty acid, amino acid and sugar metabolism in these organelles.^17^, ^31^ Placental mitochondrial dysfunction may be associated with a series of pregnancy disorders, including PE.^35^ To further investigate the possible biological functions of these acetylated proteins, we performed an enrichment analysis of GO categories (Figure 4E). The cellular component analysis revealed that DAPs were abundantly enriched in the cytoskeleton, cytoplasm and mitochondrion. DAPs were related to cytoskeletal protein binding, structural constituent of cytoskeleton, complement binding and acetyl‐CoA C‐acyltransferase activity within the molecular function category. For biological process, DAPs were involved in mitochondrial transport, regulation of cytoskeletal organization, leucocyte mediated immunity and positive regulation of vasculature development.

### 3.6 Protein–protein networks of DAPs

**FIGURE 5:**
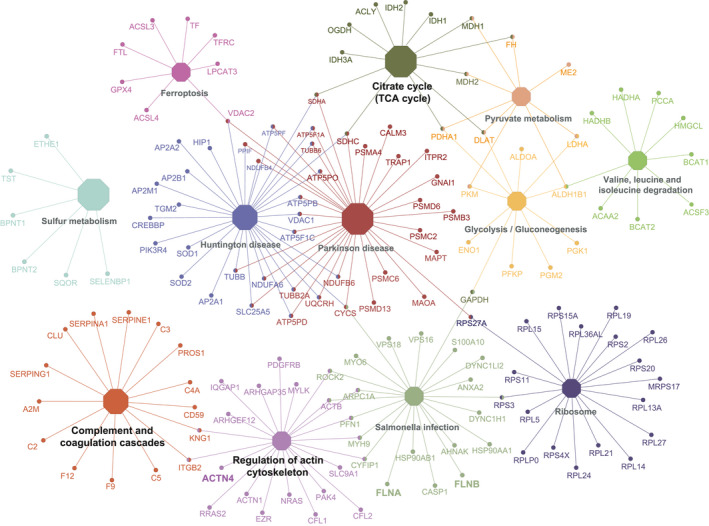
Protein–protein interaction network. Circles denotes DAPs, and different colours indicate different KEGG pathways. The mixed colour in the circle means that DAPs are enriched in multiple pathways. The size of the polygon corresponds to *p*‐value of KEGG pathway enrichment (larger polygons represent smaller *p*‐value). Abbreviations: DAPs, differentially acetylated proteins

**FIGURE 6:**
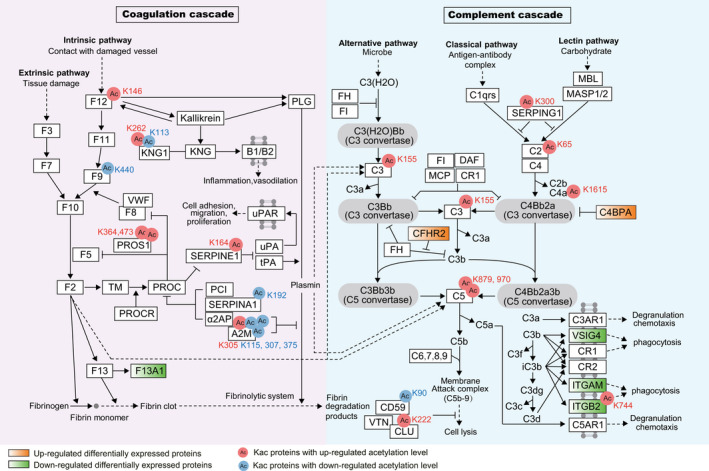
Diagram of changes in protein expression and acetylation levels between PE and NC placental tissues in complement and coagulation cascades (extracted from the KEGG database, hsa04610)

To fully describe protein function, understanding the protein–protein interaction (PPI) is very important.^36^ Here, we designed a PPI network using Cytoscape.^29^ The protein accession IDs of all the DAPs were directly uploaded to the ClueGO plugin in simple text format. Finally, 12  significantly enriched pathways were displayed (only showing pathways with <0.05). As shown in Figure 5, DAPs were involved in complement and coagulation cascades, regulation of actin cytoskeleton and citrate cycle (TCA cycle). Little is known about the relationship between other pathways and PE. Studies have reported that the activation of complement system and coagulation cascades are closely related to the pathogenesis of PE.^37^, ^38^, ^39^ To investigate whether Kac can participate in the progression of PE by affecting the complement and coagulation cascades, we further analysed DAPs in this pathway, of which 15 DAPs between PE and NC showed 21  Kac sites, including14 up‐regulated Kac sites and 7 down‐regulated Kac sites (Figure 6).

### 3.7 Verification of the potential biomarkers in PE by PRM analysis

**FIGURE 7:**
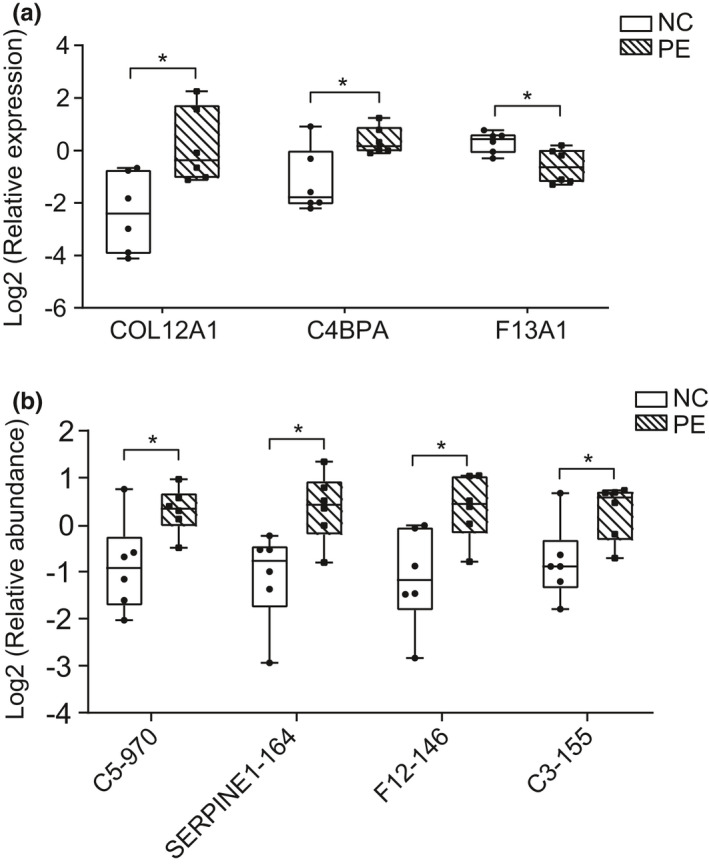
Parallel reaction monitoring (PRM) analysis. (A) PRM analysis of DEPs in proteome. The box plot shows that the relative expression levels of COL12A1 and C4BPA are significantly up‐regulated, and F13A1 is significantly down‐regulated between PE and NC (*p < 0.05). (B) PRM analysis of Kac site in acetylome. The box plot shows that the relative abundance of the acetylation peptides containing C5‐K970, SERPINE1‐K164, F12‐K146 and C3‐K155 is significantly up‐regulated in PE compared with NC (*p < 0.05)

PRM is a "targeted" mass spectrometry technique capable of verifying many targeted proteins and has been used in recent years to assess quantitative differences between biological samples.^26^, ^40^ Previous studies have utilized the LFQ‐PRM approach to identify and validate ovarian cancer‐related protein changes in patient urine samples to search for urine biomarkers.^41^ Here, we performed PRM‐based quantitative analysis of placental tissues from six women with PE and six normal‐term pregnancies to verify the results of label‐free quantification proteomics. Based on literature reports and the functions of proteins in proteomics, we performed PRM verification on several proteins and Kac sites of particular concern. The PRM results were basically consistent with LFQ data, indicating the reliability of proteomics data (Figure S1). The relative expression and abundance of the three proteins and four Kac sites were shown in the boxplot (Figure 7). Consistent with LFQ data (Figure 1B, Figure 4B), the PRM analysis results showed that COL12A1, C4BPA, C5‐K970, SERPINE1‐K164, F12‐K146 and C3‐K155 were significantly up‐regulated, and F13A1 was significantly down‐regulated in PE compared with NC. The heterogeneity of the human population explains the observed dispersion and makes the detection of biomarkers difficult.

## 4 DISCUSSION

Research has shown that PTMs are ubiquitous and play a vital role in various cellular functions. Although PTMs have been performed in a wide diversity of organisms, the function of Kac in women with PE remains poorly understood. Kac is a common, reversible and highly regulated posttranslational modification of proteins that involves many biological functions.^42^ We combined label‐free quantification proteomics and PRM analysis to investigate the characteristics of Kac in PE and revealed its potential significance.

Proteomics has provided some insights into the pathogenesis of PE. For instance, Mary et al. used gel‐free proteomic techniques to compare placental proteomes of patients with normotensive and PE, and found that DEPs were involved in various physiological processes, such as angiogenesis, oxidative stress and placental development.^43^ In another study, researchers used label‐free proteomics to identify and quantify placental proteins in a rat model of spontaneous hypertension and gestational hypertension, and found that these proteins were associated with inflammation and trophoblast invasion.^44^ Here, we found that DEPs were distributed in mitochondria and involved in multiple biological processes associated with immunity and angiogenesis. Simultaneously, most DAPs were related to the structural constituent of the cytoskeleton and complement binding, and participated in the regulation of actin cytoskeleton, complement and coagulation cascades pathways.

Mitochondrial dysfunction may exaggerate the role of innate immune response in PE, and components of the immune system may interact with angiogenic and antiangiogenic factors.^10^, ^11^ The pivotal regulator of foetal placental endothelial cell migration and angiogenesis is FLT1, which exerts antiangiogenic activity by inhibiting the signal transduction of proangiogenic factors.^45^, ^46^ Previous studies have reported that early complement activation of the placenta may stimulate sFLT1 increasing and promote the development of PE.^47^ Neutrophils dominate the effector cells of the immune system under hypoxic or inflammation conditions. The activation of neutrophils produces elastase, which may lead to vascular injury and is closely related to vascular dysfunction in PE.^11^ In our research, ITGB2 and ITGAM participated in a variety of biological processes related to immunity. Related research has reported that ITGB2 and ITGAM genes may be closely related to the pathogenesis of PE and are considered as new biomarkers of PE, which may be used for PE diagnosis.^48^, ^49^ To sum up, the immune system and angiogenesis may affect the pathogenesis of PE. Strikingly, collagen type XII alpha 1 chain (COL12A1) has the greatest fold change between PE and NC, which was an interesting finding in this study. Increased DNA methylation and gene expression at COL12A1 are associated with elevated maternal blood pressure during pregnancy, as reported earlier.^50^ Furthermore, additional research supports that COL12A1 gene is up‐regulated in PE placental tissue.^51^ Importantly, hypertension is a major characteristic of PE, which suggests that COL12A1  may be a potential candidate biomarker for the assessment of PE.

PE arises from abnormal placentation, which is associated with insufficient cytotrophoblast (CTB) cells fusion, syncytiotrophoblast (STB) dysfunction and inadequate trophoblast invasion.^52^, ^53^ In addition, the differentiation of CTB is also related to changes in cytoskeletal organization and adhesion structures, indicating that cytoskeletal integrity and appropriate remodelling may affect trophoblast cell invasion.^54^ ACTN4, an actin binding protein, is mainly expressed in placental CTB cells and participated in actin cytoskeleton dynamics and motility.^54^, ^55^ Previous evidence suggested that ACTN4 dysregulation may lead to compromised invasiveness in trophoblasts and abnormal cytoskeletal remodelling by inhibiting AKT phosphorylation, which may ultimately contribute to PE.^54^, ^55^ Filamins (FLNs) as scaffolds for signalling proteins and adhesive receptors that regulate actin cytoskeleton remodelling.^56^ At present, three members of the filament protein family, namely FLNA, FLNB and FLNC, have been discovered.^56^, ^57^ FLNA is involved in the anchoring of membrane proteins for the actin cytoskeleton.^58^ Recent studies have found that the ratio of FLNA mRNA and protein expression levels was reduced in the PE group relative to the control group.^57^, ^58^ Wei et al. suggested that trophoblastic cells invasion in PE is analogous to the behaviour of tumour cells, and the role of FLNB in PE may be similar to that in tumour progression.^59^ In addition, they also found that FLNB was mainly expressed in trophoblastic cells, and its mRNA and protein expression were down‐regulated in the PE placenta.^59^ Here, we reported for the first time that the relative abundance of multiple Kac sites of ACTN4, FLNA and FLNB were down‐regulated in the PE group compared with the NC group. Current evidence indicated that Kac of actin‐binding proteins might be involved in the pathogenesis of PE, but the specific mechanism remains further investigated.

The complement system is an integral part of innate immunity. Improper or excessive activation of the placental complement system may lead to placental dysfunction, suggesting that complement system regulation may be a potential therapeutic target for preventing PE.^38^ Notably, the most direct physical link between complement and coagulation cascade is the interaction between complement regulatory protein C4B binding protein (C4BP) and anticoagulant vitamin K‐dependent protein S (PROS1).^60^ In the early pathway of complement activation, complement factor H (CFH) and C4B binding protein (C4BP) are critical soluble regulators.^61^ CFH, a cofactor of complement factor I (CFI), competes with CFB and binds to C3B, promoting the dissociation of C3 convertase. Simultaneously, activation of C2 and C4  leads to the formation of C3 convertase and the activation of C3, which is a core component of the complement system.^61^ It has been found that C4BP deposits in the glomerulus subendothelium in patients with PE, suggesting that complement activation is associated with endothelial dysfunction in PE.^62^ Although the relationship between C4BPA and PE has not been clarified, current results found that the expression of C4BPA was significantly up‐regulated in PE, which may be a new potential biomarker for PE. Clusterin (CLU) is associated with compensation mechanisms, such as complement‐mediated inhibition of cell damage. Cell damage caused by hypoxia stress in the placenta may increase CLU expression, which plays a vital role in the pathogenesis of PE.^63^ F12 is a coagulation factor that plays a role in activating the coagulation pathway, which may be a potential pathological source of serious pregnancy complications (like PE) in gestational diabetes mellitus.^64^ Coagulation factor XIII (F13) is a transglutaminase that is essential for maintaining pregnancy.^65^ Epiney et al.^66^ used proteomics to analyse the proteins related to PE secreted by CTB cells and found that F13A1 was significantly decreased in PE CTB cells compared to the control, suggesting that the defect of coagulation factor XIII A chain (F13A1)  may be related to the development of PE. Consistent with previous studies, we found that the expression of F13A1 was significantly down‐regulated in PE placental tissues, demonstrating that F13A1  may participate in the physiopathology of PE. Previous studies used iTRAQ proteomics to assess serum and urine protein profiles in patients with PE, and found that DEPs in PE was involved in the complement system and coagulation cascades pathway.^39^, ^67^ In the present study, there were some novel discoveries that 15 DAPs in PE were involved in the complement and coagulation cascades pathway. We hypothesize that these DAPs may participate in the occurrence and development of PE by influencing the complement and coagulation cascades pathway, although no evidence has been reported to support or deny this hypothesis.

However, there were some limitations to consider in the current study. The generalizability of our findings may be limited by the current sample size. Although the results seem convincing, these findings need to be confirmed in larger prospective studies. In addition, the sample of early**‐**onset PE and late**‐**onset PE should be further separated to explore the distinction between the two phenotypes of PE.

## 5 CONCLUSION

In this study, systematic analysis of Kac revealed the critical features of placental proteins between women with PE and NC. The key features of DEPs and DAPs indicate that PE is a complex disorder connected with multiple proteins and signalling pathways. In addition, the current results have identified several new proteins in placental tissue that may have potential value in the clinical diagnosis of PE. These findings offer new insights into the complexity of PE, and the comprehensive analysis will lay the foundation for future research on the pathogenesis of PE.

## CONFLICT OF INTEREST

The authors confirm that they have no conflicts of interests.

## AUTHOR CONTRIBUTIONS

**Yu Shangguan:** Software (equal); Writing‐original draft (lead); Writing‐review & editing (equal). **Yinglan Wang:** Conceptualization (equal); Methodology (equal). **Wei Shi:** Conceptualization (equal); Methodology (equal). **Ruonan Guo:** Formal analysis (equal); Supervision (equal). **Zhipeng Zeng:** Formal analysis (equal); Supervision (equal). **Wenlong Hu:** Formal analysis (equal); Supervision (equal). **Wanxia Cai:** Investigation (equal); Resources (equal). **Qiang Yan:** Investigation (equal); Resources (equal). **Yong Xu:** Investigation (equal); Resources (equal). **Donge Tang:** Funding acquisition (equal); Project administration (equal); Visualization (equal); Writing‐review & editing (equal). **Yong Dai:** Funding acquisition (equal); Project administration (equal); Visualization (equal); Writing‐review & editing (equal).

## Supporting information

Figure S1

Data

## Data Availability

The raw data have been deposited in the OMIX (https://ngdc.cncb.ac.cn/omix) under the accession number OMIX546.
