# Supplementary material for: Systematic proteomics analysis of lysine acetylation reveals critical features of placental proteins in pregnant women with preeclampsia
Source: J Cell Mol Med. 2021 Oct 26;25(22):10614–26. doi: 10.1111/jcmm.16997 (PMC8581308; doi:10.1111/jcmm.16997)
Supplement: Supplementary file 1 — Figure S1 [file JCMM-25-10614-s001.docx]

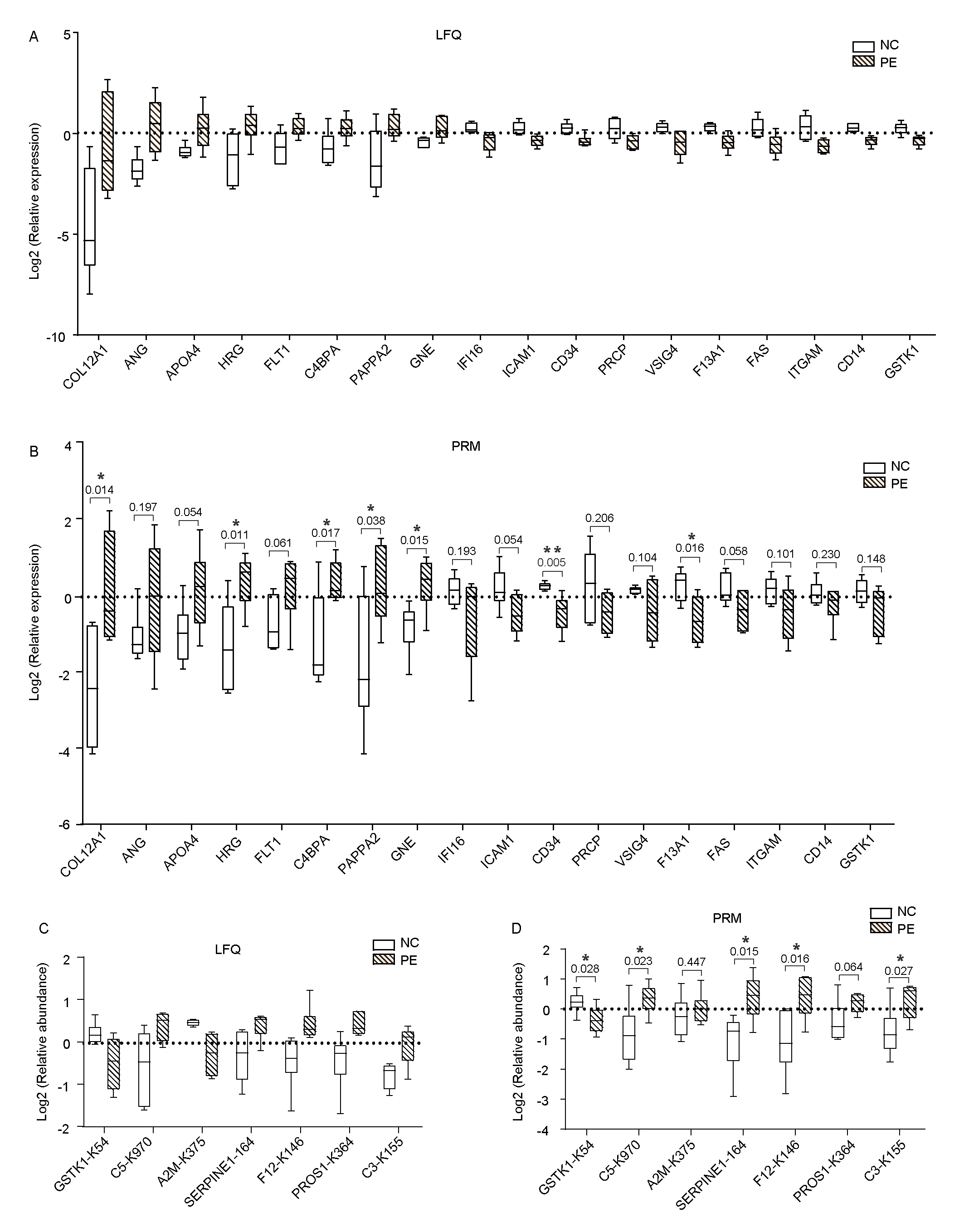


**Supplementary Figure** Box plot summarizes the relative expression of 18 DEPs and 7 Kac sites selected from proteome and acetylome, respectively. The relative expression of selected DEPs between PE and NC are depicted based on LFQ (A) and PRM analysis (B). The results of LFQ (C) and PRM analysis (D) show that the relative abundance of the 7 Kac sites between PE and NC are consistent. Abbreviations: DEPs, differentially expressed proteins; PE, preeclampsia; NC, normal-term pregnancy; LFQ: label-free quantification proteomics; PRM: parallel reaction monitoring. *P<0.05; **<0.01.
